# KRT8 Serves as a Novel Biomarker for LUAD and Promotes Metastasis and EMT *via* NF-κB Signaling

**DOI:** 10.3389/fonc.2022.875146

**Published:** 2022-05-19

**Authors:** Hao Chen, Xiaobin Chen, Bo Pan, Chutian Zheng, Liangjie Hong, Weili Han

**Affiliations:** ^1^Department of Lung Transplantation and General Thoracic Surgery, The First Affiliated Hospital, College of Medicine, Zhejiang University, Hangzhou, China; ^2^Key Laboratory of Pulsed Power Translational Medicine of Zhejiang Province, Hangzhou, China

**Keywords:** KRT8, LUAD, metastasis, EMT, NF-κB signaling

## Abstract

Keratin 8 (KRT8) is the major component of the intermediate filament cytoskeleton and aberrant expression in multiple tumors. However, the role of KRT8 in lung adenocarcinoma (LUAD) remains unclear. In the present study, KRT8 expression was found to be upregulated along with prognosis and metastasis in LUAD. Kaplan–Meier analysis presented that the 5-year OS and DSS rates were significantly better among patients with low KRT8 expression compared to those with high expression. Correlation analysis showed that KRT8 expression was significantly associated with gender (P = 0.027), advanced T stage (P = 0.001), advanced N stage (P = 0.048), and advanced pathologic stage (P = 0.025). Univariate Cox analysis demonstrated that KRT8 was a predictor of OS [hazard ratio (HR) = 1.526; 95% confidence interval (CI) 1.141–2.040; P = 0.004] and DSS (HR = 1.625; 95% CI 1.123–2.353; P = 0.010) in the TCGA database. Importantly, downregulation of KRT8 obviously suppressed cell proliferation, cell migration, invasion, and EMT as well as induced cell apoptosis. KRT8 knockdown significantly inhibited NF-κB signaling, suggesting a potential mechanism. Overall, our results indicated that KRT8 could regulate lung carcinogenesis and may serve as a potential target for antineoplastic therapies.

## Introduction

As one of the deadliest cancers worldwide, lung cancer is the second most commonly diagnosed cancer and the leading cause of cancer death in 2020 (1). In 2021, about 235,760 new cases of lung cancer will be diagnosed in the United States, and 131,880 people may die because of lung cancer (2). Lung cancer is a heterogenous disease with vastly different clinicopathological features (3), which is broadly classified as NSCLC (the majority) or SCLC (the minority). Adenocarcinoma (LUAD) is the most common subtype within NSCLC (4). The incidence of LUAD has substantially decreased, partly due to reductions in smoking rates in developed countries (5). Treatment of NSCLC is focused on surgery, chemotherapy, and radiotherapy (6). In the past decades, identification of targetable alteration in patients with NSCLC has improved its treatment (7). The approval and application of agents targeting these alterations has contributed to the decline in incidence-based mortality (8); however, there are still significant challenges as disease progression during treatment in most of these patients. Therefore, the underlying molecular mechanisms of lung cancer progression and metastasis remain to be elucidated.

Keratin 8 (KRT8) is a member of the type II keratin family clustered on the long arm of chromosome 12. KRT8 is the major component of the intermediate filament cytoskeleton and mainly expressed in simple epithelial tissues (9). KRT8 plays an important role in normal lung development and lung disease. KRT8 is a benign in epithelial cell differentiation of lung cystic fibrosis (10). In the bleomycin lung injury model, krt8^+^ transitional stem cells have squamous morphology, feature p53, and NF-κB activation (11). KRT8 is aberrantly expressed in several cancers and may serve as a biomarker (12). KRT8 is upregulated in clear cell renal cell carcinoma (13), gastric cancer (9), breast cancer (14), and lung cancer (15, 16). Aberrantly high expression of KRT8 has been found to be associated with multiple tumor progressions including cell migration (9, 13, 15, 17, 18). However, the role and mechanisms of these KRT8-mediated processes in LUAD have not been fully clarified.

In this study, we show that KRT8 was upregulated in LUAD tissues both in mRNA and protein level. The high expression of KRT8 promoted EMT and migration of lung cancer cells and was positively related with poor survival of patients with LUAD. Knockdown of KRT8 inhibited cell migration, invasion, and EMT. Moreover, these results suggest that KRT8 may regulate its biological functions through NF-κB pathway. Overall, our results indicate that KRT8 could regulate lung carcinogenesis and may serve as a potential target for antineoplastic therapies.

## Methods

### Patients in TCGA and GSE39582 Database

The data from The Cancer Genome Atlas (TCGA) database was used to analyze the KRT8 mRNA expression in patients with LUAD and its relationship in clinicopathologic features of patients with LUAD, overall survival (OS), and disease-specific survival (DSS). Then, the aberrant expression of KRT8 was confirmed using another public database, GSE116959. Follow-up was finished on April 27, 2016, on the TCGA database.

### Patients and Samples

In the present study, patients’ sample were collected with informed consent from all patients and approved by the Ethics Committee of The First Affiliated Hospital, School of Medical, Zhejiang University. Six pairs of fresh tumor tissues and adjacent normal tissues were collected and stored at −80°C or fixed in 4% paraformaldehyde overnight and paraffin-embedded for further research.

### Cell Culture

Two human lung adenocarcinoma cell lines (HCC827 and H1975) and a mice lung cancer cell line (LLC) were purchased from the Shanghai Cell Bank at the Chinese Academy of Sciences and studied in this study. Cells were cultured in alpha-Minimum Essential Medium (BI industry, Israel) supplemented with 10% fetal bovine serum (Gemini, California, USA) at 37°C in a humidified incubator with 5% CO_2_.

### Short Hairpin RNA and Plasmid Transfection

KRT8 shRNA and vector were purchased from Sangon (Beijing, China). HCC827, H1975, and LCC cells were infected with shRNA or vector with polybrene (8 μg/ml; Sigma, USA). After 48 h after infection, KRT8 knockdown efficiency was analyzed by Western blot. The sequences of shRNA are as follows: 1#: CCGGGAGCACTTGGAGAAG and 2#: CAACAAGTTTGCCTCCTTCAT.

### Western Blot

Western blot was performed as previously described (19). Antibodies used for Western blot are listed as follows: KRT8 was purchased from Boster (Wuhan, China) and used at 1 mg/ml. E-cadherin (1:1,000), N-cadherin (1:1,000), Vimentin (1:1,000), MMP2 (1:1,000), slug (1:1,000), and NF-kB (1:1,000) Signaling Pathway Antibody Sampler Panel were purchased from Abcam (UK). GAPDH (1:5000) or Lamin B1 (1:1000) were served as internal control.

### Cell Viability Assay

The Cell-Counting kit-8 (CCK-8) and colony formation assay were used to measure cell viability after KRT8 shRNA transfection. For CCK-8 assay, 5 × 10^3^ cells were implanted into 96-well plates and cultured. Then, CCK-8 assay was performed at 24, 48, 72, and 96 h according to the manufacturer’s instructions with an ELx800 absorbance microplate reader (BioTek, USA) at 450 nm. For colony formation assay, 1 × 10^3^ cells were seeded into six-well plates and cultured for another 10 days. Then, the cells were stained with crystal violet, photographed, and counted.

### Cell Apoptosis Assay

Cell apoptosis was measured by Annexin V–FITC Apoptosis Detection Kit (Dojindo, Japan) according to the manufacturer’s instructions. After trypsinization, cells were stained with annexin V and propidium iodide at room temperature for 15 min. Then, the cells were measured by flow cytometry and analyzed using FlowJo (Tree Star).

### Immunohistochemistry

Immunohistochemistry was performed as previously described (19). Antibodies used for Western blot are listed below: E-cadherin (1:200), N-cadherin (1:200), and Vimentin (1:200) were purchased from Abcam (UK).

### Transwell Migration Assay

Transwell migration assay was performed using Transwell chambers (12-μm pore size; Corning, USA). Cells (1 × 10^5^) in 200 μl of serum-free medium were seeded in the upper chambers, and 1,000 μl of medium containing 10% fetal bovine serum was added to the lower chambers to induce cell migration. After incubation for another 48 h, cells that migrated to the lower surface of the upper chambers were fixed and stained with crystal violet and pictured.

### Transwell Invasion Assay

Transwell invasion assay was performed using Transwell chambers (12-μm pore size; Corning, USA). The Matrigel matrix (Corning, USA) and serum-free medium were mixed (volume ratio of 1: 5), and then, the mixed mixture (40 μl) was added into the upper chamber of Transwell. After solidification, 1 × 10^5^ cells in 200 μl of serum-free medium were seeded in the upper chambers, and 1,000 μl of medium containing 10% fetal bovine serum was added to the lower chambers to induce cell migration. After incubation for another 48 h, cells that migrated to the lower surface of the upper chambers were fixed and stained with crystal violet and pictured.

### Xenograft Model* In Vivo*

Six-week-old male nude mice (Shanghai SLAC Laboratory Animal Co., Ltd, China) were purchased for the xenograft assay. LCC cells with control shRNA or KRT8 shRNAs were used (six mice in each group). A total volume of 0.1 ml of PBS containing 1 × 10^6^ LCC cells was injected into intravenous tail vein. Approximately 4 weeks later, tumors were observed and collected for further research. The animal study protocol was reviewed and approved by the Animal Care Committee of Zhejiang University and designed in accordance with the Interdisciplinary Principles and Guidelines for the Use of Animals in Research, Testing, and Education by the New York Academy of Sciences, *Ad Hoc* Animal Research Committee.

### Statistical Analysis

Values were presented as the mean ± SD. Experimental data were analyzed by GraphPad PRISM 6.01 software (San Diego, CA, USA). P < 0.05 indicates a statistically significant difference. * P < 0.05, ** P < 0.01, and *** P < 0.001.

## Results

### KRT8 Expression Is Upregulated in LUAD

**Figure 1:**
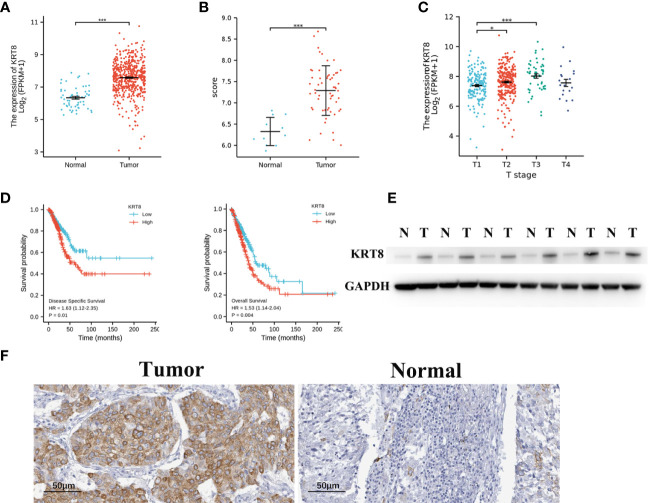
KRT8 is upregulated in LUAD and correlated with poor clinicopathologic features in LUAD. **(A)** KRT8 mRNA expression analysis in LUAD tissues (n = 535) and normal tissues (n = 59) by TCGA. **(B)** KRT8 mRNA expression analysis in LUAD tissues (n = 57) and normal tissues (n = 11) by GSE 116959. **(C)** mRNA expression of KRT8 in different stages of LUAD from TCGA. **(D)** The 5-year DSS (left) and OS (right) rate of patients with LUAD with high or low mRNA expression of KRT8 from TCGA. **(E)** KRT8 protein expression in LUAD tissues and paired para-cancer tissues (n = 6). **(F)** Representative immunohistochemical images of KRT8 expression in LUAD tissues and normal controls (×400) (Student’s t-test, *P < 0.05 and ***P < 0.001).

The TCGA and GSE116959 databases were used to examine the expression of KRT8 in LUAD. The results from TCGA indicated that the expression of KRT8 was significantly higher in cancer tissues compared to normal tissues (P < 0.001; **Figure 1A**), which is similar with the results from the GSE116959 database (P < 0.001; **Figure 1B**). Interestingly, the expression of KRT8 was moderately increased from stage I to stage III (**Figure 1C**). Further, the protein level of KRT8 was measured using patients’ samples by Western blot and IHC. Consistently, the protein expression of KRT8 was obviously upregulated in cancer tissues compared to paired normal tissues (**Figures 1E, F**). These results suggested that the upregulated KRT8 may play a vital role in the initiation and progression of LUAD.

### KRT8 Expression Correlated With Poor Clinicopathologic Features in LUAD

We then divided the TCGA cohorts into low- and high-expression subgroups according to the median expression of KRT8 to determine the significance of KRT8 in LUAD. The results from KM survival analysis indicated that both the 5-year OS and DSS rates were significantly better among patients with low KRT8 expression compared to those with high expression (P < 0.001; **Figure 1D**). Correlation analysis showed that KRT8 expression was significantly associated with gender (P = 0.027), advanced T stage (P = 0.001), advanced N stage (P = 0.048), and advanced pathologic stage (P = 0.025). No correlation was found between KRT8 expression and other clinicopathologic features (**Table 1**). Univariate Cox analysis demonstrated that KRT8 was a predictor of OS [hazard ratio (HR) = 1.526; 95% confidence interval (CI) 1.141–2.040; P = 0.004] and DSS (HR = 1.625; 95% CI 1.123-2.353; P = 0.010) in the TCGA database (**Tables 2** and **3**).

**Table 1:** Association between KRT8 expression and clinicopathologic features in the TCGA cohort of LUAD.

Characteristic	Low expression of KRT8 (n = 267)	High expression of KRT8 (n = 268)	p
T stage, n (%)			0.010
T1	104 (19.5%)	71 (13.3%)	
T2	134 (25.2%)	155 (29.1%)	
T3	18 (3.4%)	31 (5.8%)	
T4	10 (1.9%)	9 (1.7%)	
N stage, n (%)			0.028
N0	185 (35.6%)	163 (31.4%)	
N1	42 (8.1%)	53 (10.2%)	
N2	27 (5.2%)	47 (9.1%)	
N3	1 (0.2%)	1 (0.2%)	
M stage, n (%)			0.594
M0	172 (44.6%)	189 (49%)	
M1	10 (2.6%)	15 (3.9%)	
Pathologic stage, n (%)			0.025
Stage I	162 (30.7%)	132 (25%)	
Stage II	56 (10.6%)	67 (12.7%)	
Stage III	32 (6.1%)	52 (9.9%)	
Stage IV	11 (2.1%)	15 (2.8%)	
Age, n (%)			0.094
≤65	117 (22.7%)	138 (26.7%)	
>65	140 (27.1%)	121 (23.4%)	
Gender, n (%)			0.027
Female	156 (29.2%)	130 (24.3%)	
Male	111 (20.7%)	138 (25.8%)	
Smoker, n (%)			0.465
No	41 (7.9%)	34 (6.5%)	
Yes	220 (42.2%)	226 (43.4%)	

**Table 2:** Univariate and multivariate Cox proportional hazards analysis of KRT8 expression and OS for patients with LUAD in the TCGA cohort.

Characteristics	Total (N)	Univariate analysis		Multivariate analysis	
		Hazard ratio (95% CI)	P-value	Hazard ratio (95% CI)	P-value
T stage (T2 & T3 & T4 vs. T1)	523	1.728 (1.229–2.431)	0.002	1.560 (0.964–2.525)	0.070
N stage (N1 & N2 & N3 vs. N0)	510	2.601 (1.944–3.480)	<0.001	1.700 (1.121–2.576)	0.012
M stage (M1 vs. M0)	377	2.136 (1.248–3.653)	0.006	0.863 (0.405–1.843)	0.704
Gender (Male vs. Female)	526	1.070 (0.803–1.426)	0.642		
Race (White vs. Asian & Black or African American)	468	1.475 (0.902–2.411)	0.121		
Smoker (Yes vs. No)	512	0.894 (0.592–1.348)	0.591		
Pathologic stage (Stage III & Stage IV vs. Stage I & Stage II)	518	2.664 (1.960–3.621)	<0.001	1.884 (1.148–3.093)	0.012
Age (>65 vs. ≤65)	516	1.223 (0.916–1.635)	0.172		
Residual tumor (R1 & R2 vs. R0)	363	3.879 (2.169–6.936)	<0.001	2.612 (1.220–5.589)	0.013
KRT8 (High vs. Low)	526	1.526 (1.141–2.040)	0.004	1.282 (0.863–1.905)	0.219

CI, confidence interval; HR, hazard ratio.

**Table 3:** Univariate and multivariate Cox proportional hazards analysis of KRT8 expression and DSS for patients with LUAD in the TCGA cohort.

Characteristics	Total (N)	Univariate analysis		Multivariate analysis	
		Hazard ratio (95% CI)	P-value	Hazard ratio (95% CI)	P-value
T stage (T2 & T3 & T4 vs. T1)	488	1.850 (1.195–2.865)	0.006	1.503 (0.810–2.789)	0.197
N stage (N1 & N2 & N3 vs. N0)	475	2.703 (1.873–3.900)	<0.001	1.910 (1.105–3.304)	0.021
M stage (M1 vs. M0)	344	2.455 (1.269–4.749)	0.008	1.114 (0.413–3.007)	0.831
Gender (Male vs. Female)	491	0.989 (0.687–1.424)	0.954		
Race (White vs. Asian & Black or African American)	445	1.151 (0.667–1.985)	0.614		
Smoker (Yes vs. No)	477	1.040 (0.602–1.796)	0.889		
Pathologic stage (Stage III & Stage IV vs. Stage I & Stage II)	483	2.436 (1.645–3.605)	<0.001	1.382 (0.697–2.744)	0.354
Age (>65 vs. ≤65)	481	1.013 (0.701–1.464)	0.944		
Residual tumor (R1 & R2 vs. R0)	331	4.743 (2.352–9.566)	<0.001	4.043 (1.542–10.602)	0.005
KRT8 (High vs. Low)	491	1.625 (1.123–2.353)	0.010	1.006 (0.603–1.679)	0.982

CI, confidence interval; HR, hazard ratio.

### KRT8 Knockdown Inhibited Cell Proliferation and Induced Cell Apoptosis *In Vitro*

**Figure 2:**
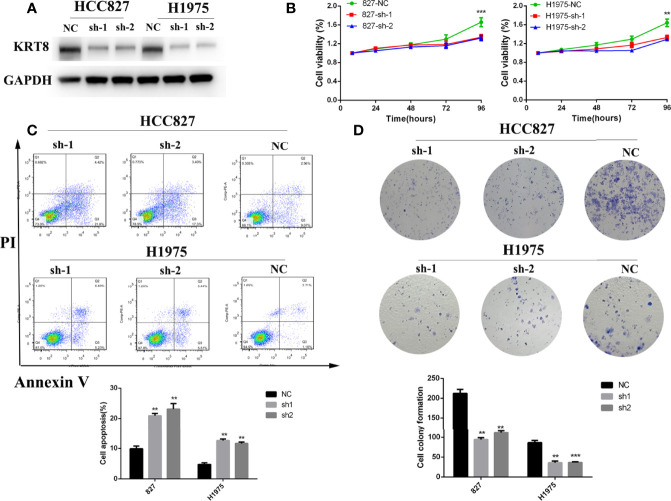
Downregulation of KRT8 inhibited cell proliferation and induced cell apoptosis in HCC827 and H1975 cells. **(A)** Evaluation of KRT8 knockdown efficiency by Western blot in HCC827 and H1975 cells after transfection with KRT8 shRNA. **(B)** Downregulation of KRT8 inhibits cell proliferation of HCC827 and H1975 cells measured by CCK-8 assay. **(C)** Downregulation of KRT8 induces cell apoptosis of HCC827 and H1975 cells. Annexin V as an assay of apoptosis. **(D)** Downregulation of KRT8 inhibits cell proliferation of HCC827 and H1975 cells measured by colony formation assay (Student’s t-test, **P < 0.01 and ***P < 0.001).

According to the relationship between KRT8 expression and clinicopathologic features, KRT8 may alter the ability of cell proliferation and apoptosis in LUAD. The CCK-8 and colony formation assay were used in two human lung cancer cell lines (HCC827 and H1975) to explore the effects of KRT8 knockdown on cell proliferation (**Figure 2A**). As shown in **Figure 2B**, CCK-8 assays showed that KRT8 knockdown inhibited the proliferation of LUAD cells. Meanwhile, the colony formation assay showed that the proliferation of lung cancer cells was suppressed by KRT8 knockdown (**Figure 2D**). Moreover, flow cytometry was applied in HCC827 and H1975 to explore the effects of KRT8 knockdown on cell apoptosis. As shown in **Figure 2C**, KRT8 knockdown obviously induced cell apoptosis of LUAD cells.

### Changed KRT8 Expression Influences Cell Migration and Invasion *In Vitro*

**Figure 3:**
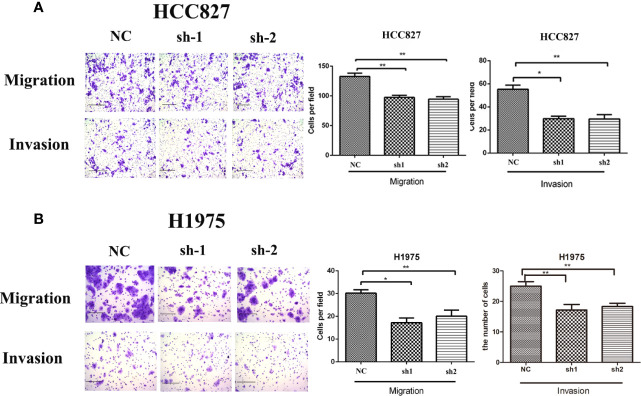
Downregulation of KRT8 reduces the migration and invasion of HCC827 and H1975 cells. **(A)** Downregulation of KRT8 reduces the migration and invasion of HCC827. **(B)** Downregulation of KRT8 reduces the migration and invasion of H1975 (Student’s t-test, *P < 0.05 and **P < 0.01).

According to the relationship between KRT8 expression and clinicopathologic features, KRT8 may also alter the ability of cell migration and invasion in LUAD. Thus, the Transwell migration and invasion assay were applied in HCC827 and H1975 to explore the effects of KRT8 knockdown on lung cancer cell motility. The results indicated that KRT8 knockdown obviously reduced the number of migrated and invaded HCC827 and H1975 cells (**Figure 3**). The expression of MMP2 was then measured by Western blot in HCC827 and H1975 cells. As shown in **Figure 6A**, KRT8 knockdown significantly downregulated the protein expression of MMP2. Overall, these data demonstrated that KRT8 knockdown could inhibit lung cancer cell migration and invasion *in vitro*.

### KRT8 Knockdown Inhibits Metastasis and EMT *In Vivo*

**Figure 4:**
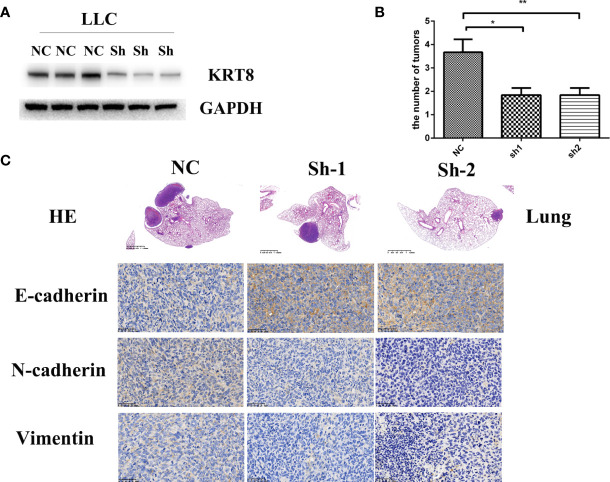
Downregulation of KRT8 inhibits the metastasis of LLC cells *in vivo*. **(A)** Evaluation of KRT8 knockdown by Western blot in LLC cells after transfection with KRT8 shRNA. **(B)** Tumor number of KRT8-knockdown and control xenografts in lung (6 mice in each group). **(C)** HE and IHC staining of KRT8-knockdown and control lung tumor samples. HE 40×, IHC of E-cadherin, N-cadherin, and vimentin 400× (Student’s t-test, *P < 0.05 and **P < 0.01).

To examine the findings *in vitro*, an *in situ* lung cancer model was built by tail intravenous injection of LLC cells. LLC cells were transfected with KRT8 shRNA or vector lentivirus and enriched by puromycin selection before injection (**Figure 4A**). After another 30 days feeding, the mice were sacrificed, and the lung was pictured and collected for Hematoxylin and eosin staining (HE) and Immunohistochemistry staining (IHC). The results showed that KRT8 knockdown efficiently inhibited the growth of tumors measured by tumor size and number (**Figure 4B**). In addition, the results from IHC indicated that KRT8 knockdown significantly downregulated N-cadherin and Vimentin but upregulated E-cadherin (**Figure 4C**). These results indicated that KRT8 could regulate metastasis and EMT of LUAD *in vivo*.

### KRT8 Expression Influences EMT *In Vitro*

**Figure 5:**
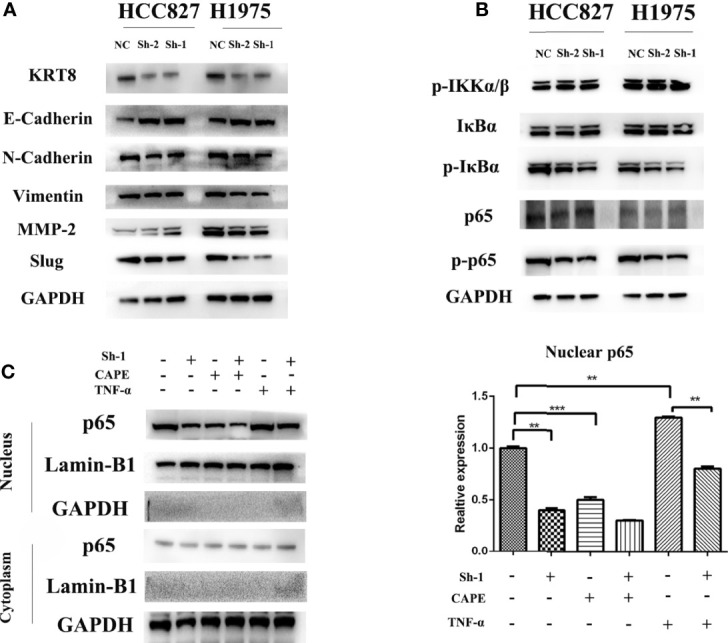
KRT8 expression affected epithelial-to-mesenchymal transition (EMT) and NF-κB signaling pathways. **(A)** EMT phenotype in KRT8 silenced lung cancer cells. Downregulation of KRT8 can reduce N-cadherin, Vimentin, MMP2, and slug expression but induce E-cadherin expression. **(B)** NF-κB signaling pathways in KRT8 silenced lung cancer cells. KRT8 knockdown downregulated the protein expression of p-IκBα and p-p65 in HCC827 and H1975 cells. **(C)** KRT8 knockdown withdraw the TNF-α–induced nuclear translocation of p65. KRT8 knockdown or treated with CAPE significantly inhibited p65 nuclear translocation, whereas those treated with TNF-α promoted the translocation (Student’s t-test, **P < 0.01 and ***P < 0.001).

EMT was measured by Western blot because of its key role in cancer cell migration and invasion. The results showed that KRT8 knockdown downregulated mesenchymal markers N-cadherin, Slug, and Vimentin but upregulated the epithelial marker E-cadherin in HCC827 and H1975 cells (**Figure 5A**). Collectively, these results indicated that KRT8 regulates the ability of migration and invasion by EMT in LUAD *in vitro*.

### KRT8-Induced EMT and Cell Motility Are Mediated by NF-κB Signaling

**Figure 6:**
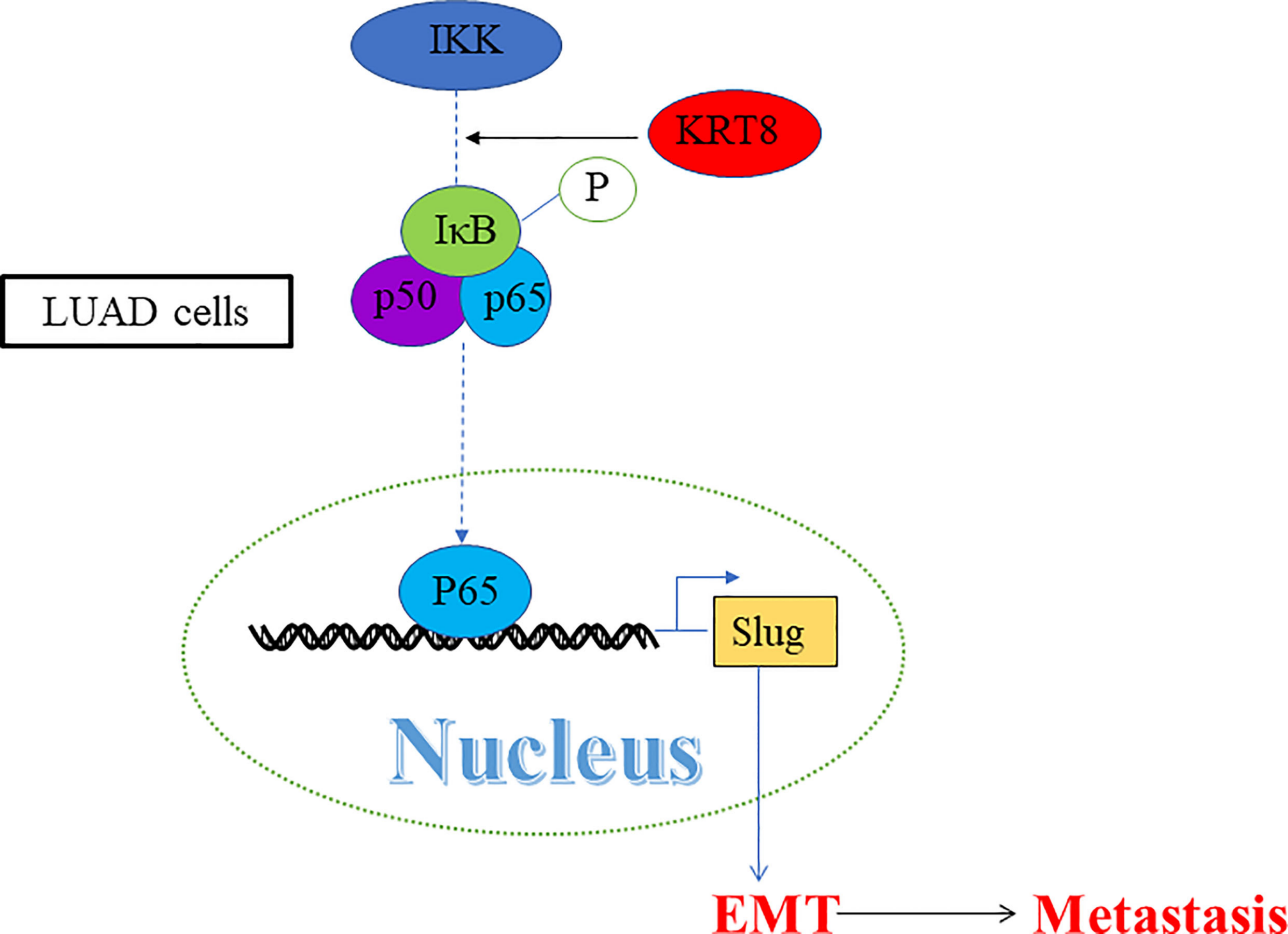
A schematic illustration showed that KRT8 promotes metastasis and EMT *via* NF-κB signaling.

Studies have already demonstrated that NF-κB pathway plays a vital role in EMT and cell motility (20). We validated the role of NF-κB pathway in KRT8-induced EMT and cell motility change. As shown in **Figure 5B**, KRT8 knockdown had no obvious effects on p-IKKα/β but significantly downregulated the protein expression of p-IκBα and p-p65 in HCC827 and H1975 cells. Moreover, p65 nuclear translocation that reflects the NF-κB activity was examined with NF-κB inducer TNF-α or inhibitor CAPE in HCC827 cells. Indeed, TNF-α promoted the baseline of p65 nuclear translocation, whereas CAPE decreased the baseline of p65 nuclear translocation. Nevertheless, KRT8 knockdown significantly inhibited p65 nuclear translocation in both TNF-α and CAPE-treated groups (**Figure 5C**). Together, KRT8-induced EMT and cell motility are mediated by NF-κB signaling in LUAD (**Figure 6**).

## Discussion

Although aberrant KRT8 expression has been studied in a variety of malignancies (9, 12–15, 17, 18), the underlying mechanisms and role of KRT8 in LUAD still remain unclear. KRT8 plays a key role in lung disease, such as lung fibrosis (10, 11). In the present study, we found that KRT8 expression was significantly increased in LUAD tissues compared to normal tissues, confirmed by data from database and our own specimens. Data analysis from TCGA indicated an important role of KRT8 in LUAD progression and is closely correlated with advanced T stage, N stage, and pathologic stage. Meanwhile, Kaplan–Meier analysis showed a negative relationship between KRT8 expression and overall and relapse-free survival of patients with LUAD. The defining feature of malignancy entails the ability to invade surrounding tissue and seed distant sites to form secondary growths (metastases). Metastatic disease is responsible for over 90% of cancer-related deaths (21). For cancer cells to occur at distant sites, these may grow into clinically relevant macrometastases (22, 23). For lung cancer, almost a third of patients develop brain metastasis at some point during their disease course (24). As reported, KRT8 plays a key role in gastric cancer (9) and renal cancer (13) by regulating cell migration and invasion. In LUAD, we found that KRT8 knockdown obviously inhibits cell migration and invasion both *in vitro* and *in vivo.* These results indicated that KRT8 may exert its function by modulating cell migration and invasion in LUAD. Epithelial-to-mesenchymal transition (EMT) is a biologic phenomenon that can alter the state of cells along a phenotypic spectrum and cause transcriptional rewiring to produce distinct tumor cell subpopulations (25). It is reported that KRT8 can participate in cell migration and invasion through EMT (18). In LUAD, the results indicated that KRT8 knockdown downregulated EMT. These results indicated that KRT8 regulates the ability of migration and invasion by EMT in LUAD.

Recent studies have demonstrated that NF-κB signaling pathway plays a vital role in oncogenesis and metastasis (26, 27). NF-κB is activated by a lot of stimulus, such as hypoxia and cytokines in tumor tissues. These stimulus activate the inhibitor of κB (IκBα) kinase (IKK) complex. Then, IKK complex leads to the phosphorylation and degradation of IκBα. IκBα is a complex made up of P50 and P65. After IκBα degradation, P50 and P65 translocate to the nucleus and regulate downstream gene expression (28). In LUAD, the results indicated that KRT8 knockdown significantly inhibits NF-κB signaling, suggesting a potential mechanism. A classical NF-κB inducer TNF-α (29) and an inhibitor CAPE (30, 31)  were used to explore the status of p65 nuclear translocation. In addition, the results indicated that KRT8 may activate NF-κB signaling *via* modulating p65 nuclear translocation. In a word, KRT8-induced EMT and cell motility are mediated by NF-κB signaling in LUAD (**Figure 6**).

In summary, our study indicates that the high expression of KRT8 regulates cell migration, invasion, and EMT by NF-κB signaling, contributing to cancer progression and poor survival in LUAD. These findings also demonstrate the potential role of KRT8 as a diagnostic and prognostic indicator in LUAD.

## Data Availability Statement

The datasets presented in this study can be found in online repositories. The names of the repository/repositories and accession number(s) can be found in the article/supplementary material.

## Ethics Statement

The studies involving human participants were reviewed and approved by the Ethics Committee of The First Affiliated Hospital, School of Medical, Zhejiang University. The patients/participants provided their written informed consent to participate in this study. The animal study was reviewed and approved by the Ethics Committee of The First Affiliated Hospital, School of Medical, Zhejiang University.

## Author Contributions

Conceptualization, HC and WH; methodology, XC; experimental investigation, BP and CZ; resources, LH and WH; writing—original draft preparation, HC, and XC; and funding acquisition, WH. All authors have read and agreed to the published version of the manuscript.

## Funding

This research was supported by the National Natural Science Foundation of China (No. 82070100), the Zhejiang Provincial Natural Science Foundation of China (No. LQ20H090003), and the medical Health Science and Technology Project of Zhejiang Provincial Health Commission (Nos. 2019RC061 and 2020KY132).

## Conflict of Interest

The authors declare that the research was conducted in the absence of any commercial or financial relationships that could be construed as a potential conflict of interest.

## Publisher’s Note

All claims expressed in this article are solely those of the authors and do not necessarily represent those of their affiliated organizations, or those of the publisher, the editors and the reviewers. Any product that may be evaluated in this article, or claim that may be made by its manufacturer, is not guaranteed or endorsed by the publisher.

## Abbreviations

KRT8, keratin8; LUAD, lung adenocarcinoma; NSCLC, non–small cell lung cancer; SCLC, small cell lung cancer; EMT, epithelial-to-mesenchymal transition; NF-κB, nuclear factor-κB; OS, overall survival; DSS, disease-specific survival; TCGA, The Cancer Genome Atlas; CCK-8, cell counting kit-8; shRNA, short hairpin RNA; PBS, phosphate-buffered saline; CAPE, phenylethyl caffeate; TNF-α, tumor necrosis factor-α.
